# Pulmonary Rehabilitation in COPD: A Reappraisal (2008–2012)

**DOI:** 10.1155/2013/374283

**Published:** 2013-01-09

**Authors:** Pierachille Santus, Linda Bassi, Dejan Radovanovic, Andrea Airoldi, Rita Raccanelli, Francesco Triscari, Francesca Giovannelli, Antonio Spanevello

**Affiliations:** ^1^Dipartimento di Scienze della Salute, Pneumologia Riabilitativa, Fondazione Salvatore Maugeri, Istituto Scientifico di Milano IRCCS, Università degli Studi di Milano, 20138 Milan, Italy; ^2^UOC di Malattie dell'Apparato Respiratorio, Azienda Ospedaliera di Desio e Vimercate, 20832 Desio, Italy; ^3^Pneumologia Riabilitativa, Fondazione Salvatore Maugeri, Istituto Scientifico di Tradate IRCCS, Università degli Studi dell'Insubria, 21049 Tradate, Italy

## Abstract

Chronic Obstructive Pulmonary Disease (COPD) is a complex pathological condition associated with an important reduction in physical activity and psychological problems that contribute to the patient's disability and poor health-related quality of life. Pulmonary rehabilitation is aimed to eliminate or at least attenuate these difficulties, mainly by promoting muscular reconditioning. The scope of this paper has been the analysis of the literature on pulmonary rehabilitation in COPD patients has appeared in the last five years, focusing on the principal outcomes obtained. The results demonstrate that pulmonary rehabilitation has a beneficial effect on dyspnoea relief, improving muscle strength and endurance. Moreover, pulmonary rehabilitation appears to be a highly effective and safe treatment for reducing hospital admissions mortality and improving health-related quality of life in COPD patients. It represents, therefore, a very important therapeutic option that, along with standard pharmachological therapy, can be used to obtain the best patient management. The favourable results obtained with pulmonary rehabilitation programs should stimulate researchers to improve our understanding of the mechanisms that form the basis of the beneficial effects of this therapeutic intervention. This would in turn increase the effectiveness of pulmonary rehabilitation in COPD patients.

## 1. Introduction 

Pulmonary rehabilitation is defined by the American Thoracic Society and the European Respiratory Society as an “evidence-based, multidisciplinary, and comprehensive intervention for patients with chronic respiratory diseases who are symptomatic and often have decreased daily life activities.” As such it is an integral part of the clinical management and health maintenance of those patients with chronic respiratory disease who remain symptomatic or continue to have decreased lung function despite standard medical treatment. Integrated into the individualised treatment of the patient, pulmonary rehabilitation is designed to reduce symptoms, optimise functional status, increase participation, and reduce health care costs by stabilising or reversing systemic manifestations of the disease [1]. All together these considerations underline the general implications and the importance of this respiratory treatment, which should be considered fundamental during the management of chronic obstructive pulmonary disease (COPD). In the last few years, medical literature has provided evidence that pulmonary rehabilitation favourably affects outcomes in COPD [2]. In spite of these important achievements, there is a need of further improvements in pulmonary rehabilitation programs, because COPD is still a major cause of disability worldwide, besides mortality [3].

COPD is characterised by complex and diverse pathophysiologic manifestations. The inflammatory pulmonary process, principally triggered by cigarette smoke, induces a series of molecular and cellular reactions with detrimental effects on lung tissue [4]. The main and more important manifestations of respiratory relevance are expiratory flow limitation with dynamic collapse of the airways, air trapping, and lung hyperinflation [5]. The increase in respiratory rate that occurs during exercise further amplifies lung hyperinflation, leading to or worsening the “dynamic hyperinflation” due to tidal expiratory flow limitation. Various bronchodilator drugs have proven able to improve pulmonary function, promoting reduction of lung hyperinflation at rest and during exercise: thus, acute administration of tiotropium or budesonide/formoterol increased inspiratory capacity and decreased intrathoracic gas volume by about 0.4 L in 20 COPD patients [6]. Helium-oxygen mixtures (heliox) are also being used to reduce lung hyperinflation in COPD patients on the assumption that if turbulent flow occurs during tidal breathing, a less dense gas mixture would reduce airway resistance and prevent expiratory flow limitation. However, heliox did not abolish expiratory flow limitation in 26 stable COPD patients but reduced exercise dynamic hyperinflation in 25% of the patients and decreased exercise dyspnoea in all of them [7]. This decrease was largely independent of changes in dynamic hyperinflation and tentatively related to the fall of inspiratory resistance which follows the reduction of turbulent flow in the upper airways with heliox.

In spite of the patient's attempt to adopt more convenient breathing patterns, these adaptations are generally overwhelmed during exercise, when there is an acute increase in the ventilatory demand. Acute and chronic hyperinflation have been shown to contribute to exertional dyspnoea, reduced ventilatory capacity, and worsened exercise performance in COPD [8, 9]. Wasted ventilation further increases the already high ventilatory demand requested for the maintenance of blood gas homeostasis. 

Although the initial pathology of COPD is confined to the lung, the reduction in physical activity and psychological problems associated to the progress of the disease increasingly contribute to the patient's disability and poor health-related quality of life. This forms the basis of the most important clinical manifestations of COPD, such as muscle dysfunction, cardiac impairment, skeletal and sensory deficits, malnutrition, and steroid-related myopathy [10], besides respiratory muscle fatigue, sleep disorders, and psychological alterations such as anxiety, depression, sense of guilt, and carer dependency. The importance of the psychological profile has been clearly demonstrated, particularly as far as anxiety and depression are concerned, both being common occurrence in COPD patients, even when their disease is mild in terms of respiratory function and symptoms [11]. Indeed, depression has a prevalence rate of about 45% in patients with moderate to severe COPD [12]. Hence, care should be taken to design an adequate psychological and social support within the pulmonary rehabilitation settings. 

Exercise training is an important aspect of pulmonary rehabilitation, as it represents the best available means of improving muscle performance, with remarkable favourable impact on exertional dyspnoea, exercise tolerance, and improvement of daily activities [1]. Traditionally pulmonary rehabilitation has focused on lower extremity training, little or no attention being paid to training of upper limb muscles, although they are regularly involved in all daily activities. The minimum duration of exercise training in pulmonary rehabilitation has not been extensively investigated; however, the ERS/ATS Statement suggests 20 sessions of a comprehensive treatment as the best option. 

Education of the patient is a core component of a complete rehabilitation program, together with the prevention and early treatment of respiratory exacerbations, implementation of breathing strategies, and bronchial cleaning. The combination of postural drainage, percussion, and forced expiration improve airway clearance, while the use of a positive expiratory pressure mask and assisted coughing have proven to be more effective than assisted coughing alone in COPD patients during an exacerbation [13]. In fact, for some patients mucus hypersecretion and impaired mucociliary transport represent distinctive features of their lung disease, and for these reasons they require particular and appropriate instructions. 

Pulmonary rehabilitation programs should also address body composition abnormalities, which are frequently present but underrecognised in chronic lung diseases. Interventions relating to these aspects may be in the form of caloric supplementation, physiological interventions, pharmacological strategies, or combination therapy in order to induce weight gain without an overall fat mass increase. All of these interventions have resulted in an improvement in quality of life and justify the decision taken by official organisations to recommend pulmonary rehabilitation as an integral part of the long-term management of COPD [14–17]. 

While the utility of pulmonary rehabilitation is undisputed, no general consensus exists regarding the parameters that should best represent the improvements achieved with pulmonary rehabilitation. Indeed, the various research groups have focused on different parameters, like exercise performance, endurance, dyspnoea, and quality of life, while little or no attention have been paid to a number of parameters concerned with respiratory mechanics and gas exchange. The absence of homogeneity regarding the study endpoints largely limits the comparison among the various studies, besides evaluation of their results. With this in mind, we have analysed the literature on pulmonary rehabilitation in COPD patients that has appeared in the last five years, focusing on the main outcomes used and their evaluation.

## 2. Selection Criteria

**Figure 1:**
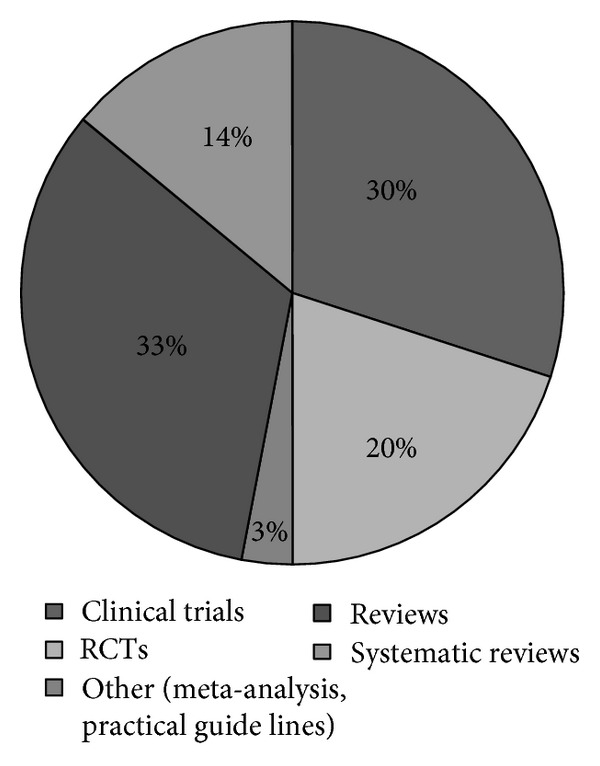
Distribution of scientific literature about pulmonary rehabilitation.

 We conducted a MEDLINE search using the keywords “pulmonary rehabilitation” and “COPD”: of the resulting 1294 articles, 574 had been published in the last five years, but only 398 had pulmonary rehabilitation as the relevant issue. These papers could be classified as follows: 121 clinical trials, 78 randomised clinical trials, 10 meta-analyses, 4 practical guide lines, 131 reviews, and 54 systematic reviews (Figure 1). Guide lines and reviews were discarded. Among clinical trials, we took into consideration those that were performed following a randomisation design and those which included a representative number of patients (>200). Two additional studies involving a smaller number of patients were included in the analysis: one because it addresses the results obtained in an out-patient pulmonary rehabilitation program [18], the other because it deals with a new approach, namely, the home exercise video program [19]. With these restrictions, only 19 papers could be taken into consideration (Table 1).

## 3. Results

In all studies, the duration of the pulmonary rehabilitation programs was six-to-twelve weeks. Furthermore, all programs were based on a multidisciplinary approach: exercise training, patient education, psychosocial and behavioural interventions, and nutritional therapy to contrast weight loss and muscle wasting. It is important to underline that only three papers made the distinction between primary and secondary outcomes. Furthermore, two of them have evaluated the improvement of the quality of life after the Saint George Respiratory Questionnaire (SGRQ) score as the primary outcome [20, 21], whereas the third one has used the Chronic Respiratory Disease Questionnaire (CRQ) [22].

The conclusion common to all papers listed in Table 1 is that pulmonary rehabilitation improves the 6 MWTD, maximal oxygen consumption, treadmill endurance time, exertional and overall dyspnoea, and self-efficacy for walking, in line with studies performed in the 90s [37–39]. Briefly, the assessment of pulmonary rehabilitation has been made according to three perspectives: functional outcomes, dyspnoea perception, and quality of life.

### 3.1. Functional Outcomes

Twelve studies have analysed the six-minute walking test distance (6 MWTD) as a functional parameter; all of them concluded that pulmonary rehabilitation of COPD patients leads to an increase of the covered distance. Nine of those papers also evaluated other variables such as the incremental shuttle test, leg strength, and the peak oxygen intake and found that they were correlated with the 6 MWTD. Moreover, in a retrospective analysis involving a cohort of 815 severe or very severe COPD patients undergoing a pulmonary rehabilitation program based on increasing exercise tolerance, transfers, and stair climbing, Enfield et al. [28] found that the 6 min walking distance was increased by an average 90 metres and that these changes were positively associated with the increase of survival rate. Therefore, the 6 MWTD appears to be an important, simple, and repeatable parameter to evaluate the functional improvement obtained with a pulmonary rehabilitation program, independent of the severity of the disease.

Only 3 studies have considered *Forced Expiratory Volume in the first second* (FEV_1_) as a functional parameter. Stav et al. [31] reported a consistent reduction of the rate of FEV_1_ decline or even a suppression of that decline after three years of pulmonary rehabilitation, while Ergün et al. [18] and Chang et al. [20] found no significant changes in FEV_1_ after 8 weeks of pulmonary rehabilitation. 

### 3.2. Dyspnoea Perception

Four studies evaluated dyspnoea using the MRC or Borg scale. Ergün et al. [18] demonstrated a decrease in dyspnoea sensation by an average of 1.2 units of the MRC scale, both in the early and the late-stage group of COPD patients. Similarly, no significant differences in dyspnoea score were observed between those two groups in a randomized, controlled, prospective study on 78 COPD patients aiming to assess the effectiveness of a pulmonary rehabilitation program performed in a community hospital [34]. On the contrary, Scott et al. [30], using the Borg scale to assess dyspnoea severity in a prospective, observational study, concluded that patients with higher baseline FEV_1_ were more likely to enjoy an attenuation of breathlessness, besides greater improvement of both subjective (SGRQ) and objective outcomes (6 MWD).

Few data are available concerning the effectiveness of pulmonary rehabilitation on dyspnoea relief in less severe COPD patients. In a 2-year randomised controlled trial on patients with moderate airflow obstruction but impaired exercise capacity, it was found that a significantly greater decrease of MRC dyspnoea score from baseline occurred in the group of patients subjected to active treatment [21].

### 3.3. Quality of Life

Ten studies have evaluated the improvements in quality of life using either the SGRQ, CRQ, or Hospital Anxiety Depression (HADs) scale. Van Wetering et al. [21] have conducted a 2-year randomised controlled trial in which the efficacy of the conventional treatments was compared with that of a newly designed Interdisciplinary Community-based (INTERCOM) COPD management program, consisting in a 4-month rehabilitation phase and a 20-month active maintenance phase. The primary outcomes were the change from baseline in disease-specific quality of life as assessed by the SGRQ total score and the total number of exacerbations. At 12 months, the SGRQ score in the INTERCOM group had almost returned to baseline, whereas in the conventional care group it remained stable up to 12 months and worsened thereafter. The authors concluded that INTERCOM proved to be a feasible approach to improve disease-specific quality of life, dyspnoea, and functional exercise capacity. On the other hand, the frequency of exacerbations was not significantly different between the groups during the 2-year period of observation. The other two studies that used quality of life as primary outcome also concluded that pulmonary rehabilitation is effective in improving the health-related quality of life in COPD patients [20, 22], and a similar conclusion was reached in the studies that have used quality of life as an additional or secondary outcome [18, 19, 23, 24, 30, 34, 35].

It should be stressed, however, that these studies were carried out on patients with a stable disease, while it is well known that exacerbations are an important and negative prognostic element in the natural history of the disease [40], becoming more frequent as the disease progresses [41]. This should be taken into account for a comprehensive evaluation of the impact of pulmonary rehabilitation programs. Indeed, randomised controlled trials performed over a 2-year period have shown that pulmonary rehabilitation has no impact on incidence of exacerbations and health-care utilization, although there were improvements in disease-specific quality of life, dyspnoea scores, and exercise capacity [21, 33]. 

## 4. Discussion 

 The purpose of this paper has been to analyse the outcomes used in studies on pulmonary rehabilitation in COPD patients published over the last five years, besides the efficacy of these treatments in improving the quality of life and the ability in carrying out daily life activities. Almost all studies have assumed as a primary outcome for the evaluation of the pulmonary rehabilitation programs the distance covered during the six-minute walking test and the peripheral muscle strength, with less attention paid to the impact on the quality of life.

A growing amount of literature advocates home-based rehabilitation as a useful adjunct for COPD management [42, 43]. Home-based interventions are a cheaper, more cost-effective method of care than traditional hospital treatment [44–46] and enable patients to remain in their own environments, close to the family, where exercise training specific to their daily activities can be applied [42, 47, 48]. It must be noted, however, that home-based interventions are principally focused on respiratory muscle training, whereas pulmonary rehabilitation performed in the hospital tackles additional aspects, such as quality of life, breathlessness sensation, psychological profile, and effectiveness of therapeutic interventions [49]. Furthermore, hospital-based, multidisciplinary pulmonary rehabilitation programs include interventions that promote airway clearance, as accumulation of secretions in COPD contributes substantially to airway obstruction. Application of positive end-expiratory pressure has been shown very effective to remove bronchial secretions and reduce atelectasis. It is also the most effective treatment available in reducing the need for increased ventilatory assistance and duration of hospital stay after an exacerbation [50], while concomitant bronchodilation therapy can also help mobilisation of secretions, as it positively affects the ciliary beat frequency of respiratory epithelium [51]. Furthermore, several studies have investigated the nonadherence to inhalatory medications of COPD patients; it has been in fact reported that to 18% of patients spontaneously discontinue the respiratory therapy [52], and it is reasonable to suppose that the incidence would be greater in patients involved in a home-based rehabilitation program. On the other hand, there is a paucity of data regarding the adherence in attending pulmonary rehabilitation programs. In a retrospective analysis, Sabit et al. [36] have concluded that COPD patients are less likely to complete a pulmonary rehabilitation program if they are current smokers, attend a long lasting program, suffer frequent exacerbations requiring hospital admission, and have higher MRC score. There is therefore the need for a worldwide multicentre investigation to better understand what kind of COPD patients should be assigned to pulmonary rehabilitation programs, also in connection with the available resources. 

Of particular interest is the hypothesis that pulmonary rehabilitation, through the exercise and nutritional intervention, could reduce the risk of cardiovascular accidents [26], because it is well known that patients with COPD have an increased risk of cardiovascular disease. The hypothesis was supported by the observation that following rehabilitation, the aortic pulse wave velocity (PWV) was reduced together with a marked fall in systemic blood pressure [26]. Moreover, there was a modest reduction in total cholesterol. This study, the first that evaluates the effect of a standardised multidisciplinary pulmonary rehabilitation program on cardiovascular risk factors in patients with COPD, indicates that pulmonary rehabilitation could represent an opportunity to identify and treat cardiovascular and metabolic dysfunction in these patients, thus providing additional benefits.

The primary goal of pulmonary rehabilitation should be, however, the improvement of lung mechanics, in order to lower the work of breathing and restore ventilation-perfusion distribution, with enhanced gas exchange and exercise performance. These pathogenetic cornerstones of COPD should be treated both with pharmacological bronchodilation and pulmonary rehabilitation, in order to reduce respiratory symptoms in stable patients and during exacerbations [51, 53]. There are, in fact, clear indications for performing pulmonary rehabilitation after acute exacerbations in COPD patients, besides conventional community care, as this treatment appears to be safe and highly effective in reducing hospital admissions and mortality and in improving health-related quality of life [54]. 

## 5. Conclusion

Current literature supports the notion that pulmonary rehabilitation provides clinically relevant improvements in quality of life, breathlessness, exercise performance, and psychological status. Also the usefulness of the association of conventional pharmacological treatment and pulmonary rehabilitation has been repeatedly proven [49]. However, uncertainties remain regarding some elements of pulmonary rehabilitation programs, such as duration and yearly frequency of the cycles, training intensity, and degree of supervision, for which further investigations are required. Furthermore, the present analysis has shown that only very few studies have considered pulmonary function parameters among expected outcomes. This, together with lack of assessment of absolute lung volume partitioning and tidal expiratory flow limitation, largely prevents the possibility to better understand the effects of pulmonary rehabilitation on the respiratory system, urging for further studies in this area.

## Figures and Tables

**Table 1:** Selected studies for the review and used outcomes.

Main author/year	Type of study	Outcomes
van Ranst et al. 2011 [23]	Retrospective, observational	(i) Peripheral muscle performance (ii) Respiratory muscle strength (iii) Cycle exercise endurance and 6 MWDT (iv) CRQ, SGRQ, SF-36

Yoshimi et al. 2012 [24]	Prospective, observational	(i) Respiratory muscle strength (ii) 6 MWDT (iii) SGRQ

Murphy et al. 2011 [22]	Single-blind cluster randomised trial	(i) 1°: CRQ (ii) 2°: ISWT, Self-efficacy for Managing Chronic Disease 6-Item Scale

Fischer et al. 2012 [25]	Prospective, observational	(i) 6 MWDT (ii) Correlation between concerns about exercise and 6 MWDT

Gale et al. 2011 [26]	Prospective cohort study	(i) PWV, BP, IL-6, fasting glucose and lipids (ii) ISWT

Riario-Sforza et al. 2009 [27]	Number needed to treat study	6 MWDT

Enfield et al. 2010 [28]	Retrospective, observational cohort study	Relationship between 6 MWD and survival

Cheikh Rejbi et al. 2010 [29]	Prospective, observational	6 MWDT and peak oxygen uptake in COPD and healthy subjects

Ergün et al. 2011 [18]	Prospective, observational	(i) MRC, BORG dyspnea scale (ii) ISWT, ESWT, FEV_1_ (iii) SGRQ, HADs (iv) Body composition: BMI, FFM

Scott et al. 2010 [30]	Prospective, observational	(i) Compliance (ii) SGRQ (iii) 6 MWDT (iv) BORG dyspnea scale

Stav et al. 2009 [31]	Matched controlled trial	(i) FEV_1_ (ii) 6 MWDT (iii) BMI

Moore et al. 2009 [19]	Randomised pilot study	(i) ISWT (ii) CRQ

Steele et al. 2008 [32]	Randomised clinical trial	(i) Daily activity with accelerometer (ii) Exercise adherence with diary (iii) 6 MWDT

Eaton et al. 2009 [33]	Prospective randomized controlled study	Risk of readmission at 3 months after an exacerbation

Chang et al. 2008 [20]	Three-group randomised controlled trial	(i) 1°: SGRQ (ii) 2°: FAI, IPAQ, ISWT, FEV_1_

Elçi et al. 2008 [34]	Randomized, controlled, prospective study	(i) MRC (ii) 6MWDT (iii) SF-36 (iv) HADs (v) SGRQ

Van Wetering et al. 2010 [21]	Randomised controlled trial	(i) 1°: SGRQ, *n *° of exacerbations (ii) 2°: subscores of SGRQ, MRC, 6 MWDT, muscle strength, FFM, lung function

Gottlieb et al. 2011 [35]	Single-centre, randomized, placebo-controlled, unblinded clinical trial	(i) 6 MWDT (ii) Leg strength (iii) SGRQ

Sabit et al. 2008 [36]	Retrospective case note study	Identifying variables that affect poor attendance to PR programme

6 MWDT: 6-minute walking distance test, CRQ: chronic respiratory disease questionnaire, SGRQ: St George's respiratory questionnaire, SF-36: medical outcomes study short-form survey, PWV: aortic pulse wave velocity, BP: blood pressure, IL-6: interleukin-6, ISWT: incremental shuttle walk test, ESWT: endurance shuttle walking test, HADs: hospital anxiety depression scale, BMI: body mass index, FFM: fat free mass, FEV_1_: forced expiratory volume in one second, FAI: Frenchay activities index, IPAQ: international physical activity questionnaire.

## References

[1] Nici L, Donner C, Wouters E (2006). American thoracic society/European Respiratory Society statement on pulmonary rehabilitation. *The American Journal of Respiratory and Critical Care Medicine*.

[2] ACCP/AACVPR (1997). Pulmonary rehabilitation guidelines panel. Pulmonary rehabilitation: joint ACCP/AACVPR evidence-based guidelines. *Chest*.

[3] Hurd S (2000). The impact of COPD on lung health worldwide: epidemiology and incidence. *Chest*.

[4] Crapo RO, Jensen RL, Hargreave FE (2003). Airway inflammation in COPD: physiological outcome measures and induced sputum. *European Respiratory Journal*.

[5] Aliverti A, Macklem PT (2008). The major limitation to exercise performance in COPD is inadequate energy supply to the respiratory and locomotor muscles. *Journal of Applied Physiology*.

[6] Santus P, Centanni S, Verga M, Di Marco F, Matera MG, Cazzola M (2006). Comparison of the acute effect of tiotropium versus a combination therapy with single inhaler budesonide/formoterol on the degree of resting pulmonary hyperinflation. *Respiratory Medicine*.

[7] D’Angelo E, Santus P, Civitillo MF, Centanni S, Pecchiari M (2009). Expiratory flow-limitation and heliox breathing in resting and exercising COPD patients. *Respiratory Physiology and Neurobiology*.

[8] O'Donnell DE, Webb KA (1993). Exertional breathlessness in patients with chronic airflow limitation: the role of lung hyperinflation. *The American Review of Respiratory Disease*.

[9] O’Donnell DE, Revill SM, Webb KA (2001). Dynamic hyperinflation and exercise intolerance in chronic obstructive pulmonary disease. *The American Journal of Respiratory and Critical Care Medicine*.

[10] Decramer M, de Bock V, Dom R (1996). Functional and histologic picture of steroid-induced myopathy in chronic obstructive pulmonary disease. *The American Journal of Respiratory and Critical Care Medicine*.

[11] Di Marco F, Verga M, Reggente M (2006). Anxiety and depression in COPD patients: the roles of gender and disease severity. *Respiratory Medicine*.

[12] Mills TL (2001). Comorbid depressive symptomatology: isolating the effects of chronic medical conditions on self-reported depressive symptoms among community-dwelling older adults. *Social Science and Medicine*.

[13] Bellone A, Spagnolatti L, Massobrio M (2002). Short-term effects of expiration under positive pressure in patients with acute exacerbation of chronic obstructive pulmonary disease and mild acidosis requiring non-invasive positive pressure ventilation. *Intensive Care Medicine*.

[14] American Thoracic Society (1999). Pulmonary rehabilitation: official statement of the American Thoracic Society Board of Directors. *The American Journal of Respiratory Critical and Care Medicine*.

[15] O'Donnell DE, Hernandez P, Aaron S (2003). Canadian Thoracic Society COPD Guidelines: summary of highlights for family doctors. *Canadian Respiratory Journal*.

[16] Pauwels RA, Buist AS, Calverley PMA, Jenkins CR, Hurd SS (2001). Global strategy for the diagnosis, management, and prevention of chronic obstructive pulmonary disease: National Heart, Lung, and Blood Institute and World Health Organization Global Initiative for Chronic Obstructive Lung Disease (GOLD): executive summary. *Respiratory Care*.

[17] Troosters T, Donner CF, J Schols AMW (2006). Rehabilitation in chronic obstructive pulmonary disease. *European Respiratory Monograph*.

[18] Ergün P, Kaymaz D, Günay E (2011). Comprehensive out-patient pulmonary rehabilitation: treatment outcomes in early and late stages of chronic obstructive pulmonary disease. *Annals of Thoracic Medicine*.

[19] Moore J, Fiddler H, Seymour J (2009). Effect of a home exercise video programme in patients with chronic obstructive pulmonary disease. *Journal of Rehabilitation Medicine*.

[20] Chang AT, Haines T, Jackson C (2008). Rationale and design of the PRSM study: pulmonary rehabilitation or self management for chronic obstructive pulmonary disease (COPD), what is the best approach?. *Contemporary Clinical Trials*.

[21] Van Wetering CR, Hoogendoorn M, Mol SJM, Rutten-Van Mölken MPMH, Schols AM (2010). Short- and long-term efficacy of a community-based COPD management programme in less advanced COPD: a randomised controlled trial. *Thorax*.

[22] Murphy K, Casey D, Devane D (2011). A cluster randomised controlled trial evaluating the effectiveness of a structured pulmonary rehabilitation education programme for improving the health status of people with chronic obstructive pulmonary disease (COPD): the PRINCE Study protocol. *BMC Pulmonary Medicine*.

[23] van Ranst D, Otten H, Meijer JW, van't Hul AJ (2011). Outcome of pulmonary rehabilitation in COPD patients with severely impaired health status. *International Journal of Chronic Obstructive Pulmonary Disease*.

[24] Yoshimi K, Ueki J, Seyama K (2012). Pulmonary rehabilitation program including respiratory conditioning for chronic obstructive pulmonary disease (COPD): improved hyperinflation and expiratory flow during tidal breathing. *Journal of Thoracic Disease*.

[25] Fischer MJ, Scharloo M, Abbink J (2012). Concerns about exercise are related to walk test results in pulmonary rehabilitation for patients with COPD. *International Journal of Behavioral Medicine*.

[26] Gale NS, Duckers JM, Enright S, Cockcroft JR, Shale DJ, Bolton CE (2011). Does pulmonary rehabilitation address cardiovascular risk factors in patients with COPD?. *BMC Pulmonary Medicine*.

[27] Riario-Sforza GG, Incorvaia C, Paterniti F (2009). Effects of pulmonary rehabilitation on exercise capacity in patients with COPD: a number needed to treat study. *International Journal of Chronic Obstructive Pulmonary Disease*.

[28] Enfield K, Gammon S, Floyd J (2010). Six-minute walk distance in patients with severe end-stage COPD: association with survival after inpatient pulmonary rehabilitation. *Journal of Cardiopulmonary Rehabilitation and Prevention*.

[29] Cheikh Rejbi IB, Trabelsi Y, Chouchene A (2010). Changes in six-minute walking distance during pulmonary rehabilitation in patients with COPD and in healthy subjects. *International Journal of Chronic Obstructive Pulmonary Disease*.

[30] Scott AS, Baltzan MA, Fox J, Wolkove N (2010). Success in pulmonary rehabilitation in patients with chronic obstructive pulmonary disease. *Canadian Respiratory Journal*.

[31] Stav D, Raz M, Shpirer I (2009). Three years of pulmonary rehabilitation: inhibit the decline in airflow obstruction, improves exercise endurance time, and body-mass index, in chronic obstructive pulmonary disease. *BMC Pulmonary Medicine*.

[32] Steele BG, Belza B, Cain KC (2008). A randomized clinical trial of an activity and exercise adherence intervention in chronic pulmonary disease. *Archives of Physical Medicine and Rehabilitation*.

[33] Eaton T, Young P, Fergusson W (2009). Does early pulmonary rehabilitation reduce acute health-care utilization in COPD patients admitted with an exacerbation? A randomized controlled study. *Respirology*.

[34] Elçi A, Börekçi S, Ovayolu N, Elbek O (2008). The efficacy and applicability of a pulmonary rehabilitation programme for patients with COPD in a secondary-care community hospital. *Respirology*.

[35] Gottlieb V, Lyngsø AM, Nybo B (2011). RehabilItation for moderate COPD (GOLD 2) does it have an effect?. *Chronic Obstructive Pulmonary Disease*.

[36] Sabit R, Griffiths TL, Watkins AJ (2008). Predictors of poor attendance at an outpatient pulmonary rehabilitation programme. *Respiratory Medicine*.

[37] Goldstein RS, Gort EH, Stubbing D, Avendano MA, Guyatt GH (1994). Randomised controlled trial of respiratory rehabilitation. *The Lancet*.

[38] Ries AL, Kaplan RM, Limberg TM, Prewitt LM (1995). Effects of pulmonary rehabilitation on physiologic and psychosocial outcomes in patients with chronic obstructive pulmonary disease. *Annals of Internal Medicine*.

[39] Wijkstra PJ, van der Mark TW, Kraan J, van Altena R, Koëter GH, Postma DS (1996). Effects of home rehabilitation on physical performance in patients with chronic obstructive pulmonary disease (COPD). *European Respiratory Journal*.

[40] Seemungal TAR, Donaldson GC, Paul EA, Bestall JC, Jeffries DJ, Wedzicha JA (1998). Effect of exacerbation on quality of life in patients with chronic obstructive pulmonary disease. *The American Journal of Respiratory and Critical Care Medicine*.

[41] Hurst JR, Vestbo J, Anzueto A (2010). Susceptibility to exacerbation in chronic obstructive pulmonary disease. *The New England Journal of Medicine*.

[42] Wijkstra PJ, Strijbos JH (1998). Home-based rehabilitation for patients with chronic obstructive pulmonary disease. *Monaldi Archives for Chest Disease*.

[43] Wijkstra PJ (2003). Home based rehabilitation for patients with COPD. Is it equally effective as compared to outpatient rehabilitation?. *Monaldi Archives for Chest Disease*.

[44] Reina-Rosenbaum R, Bach JR, Penek J (1997). The cost/benefits of outpatient-based pulmonary rehabilitation. *Archives of Physical Medicine and Rehabilitation*.

[45] Wijkstra PJ, Strijbos JH, Köter GH (2000). Home-based rehabilitation for patients with COPD: organization, effects and financial implications. *Monaldi Archives for Chest Disease*.

[46] Hernandez C, Casas A, Escarrabill J (2003). Home hospitalisation of exacerbated chronic obstructive pulmonary disease patients. *European Respiratory Journal*.

[47] Wijkstra PJ, van der Mark TW, Kraan J, van Altena R, Koëter GH, Postma DS (1996). Long-term effects of home rehabilitation on physical performance in chronic obstructive pulmonary disease. *The American Journal of Respiratory and Critical Care Medicine*.

[48] Ambrosino N, Strambi S (2004). New strategies to improve exercise tolerance in chronic obstructive pulmonary disease. *European Respiratory Journal*.

[49] Lacasse Y, Goldstein R, Lasserson TJ, Martin S (2009). Pulmonary rehabilitation for chronic obstructive pulmonary disease (review). *The Cochrane Library*.

[50] Osadnik CR, McDonald CF, Jones AP (2012). Airway clearance techniques for chronic obstructive pulmonary disease (review). *Cochrane Database of Systematic Reviews*.

[51] Piatti G, Ambrosetti U, Santus P, Allegra L (2005). Effects of salmeterol on cilia and mucus in COPD and pneumonia patients. *Pharmacological Research*.

[52] Santus P, Picciolo S, Proietto A (2012). Doctor-patient relationship: a resource to improve respiratory diseases management. *European Journal of Internal Medicine*.

[53] Cazzola M, Santus P, Di Marco F (2003). Bronchodilator effect of an inhaled combination therapy with salmeterol plus fluticasone and formeterol plus budesonide in patients with COPD. *Respiratory Medicine*.

[54] Puhan MA, Gimeno-Santos E, Scharplatz M, Troosters T, Walters EH, Steurer J (2011). Pulmonary rehabilitation following exhacerbations of chronic obstructive pulmonary disease (review). *The Cochrane Library*.

